# Investigating poultry trade patterns to guide avian influenza surveillance and control: a case study in Vietnam

**DOI:** 10.1038/srep29463

**Published:** 2016-07-12

**Authors:** Guillaume Fournié, Astrid Tripodi, Thi Thanh Thuy Nguyen, Van Trong Nguyen, Trong Tung Tran, Andrew Bisson, Dirk U. Pfeiffer, Scott H. Newman

**Affiliations:** 1 Veterinary Epidemiology, Economics and Public Health Group, Department of Production and Population Health, Royal Veterinary College, University of London, United Kingdom; 2 Emergency Center for the Control of Transboundary Animal Diseases (ECTAD), Food and Agriculture Organization of the United Nations (FAO), Hanoi, Vietnam; 3 Department of Livestock Production, Hanoi, Vietnam

## Abstract

Live bird markets are often the focus of surveillance activities monitoring avian influenza viruses (AIV) circulating in poultry. However, in order to ensure a high sensitivity of virus detection and effectiveness of management actions, poultry management practices features influencing AIV dynamics need to be accounted for in the design of surveillance programmes. In order to address this knowledge gap, a cross-sectional survey was conducted through interviews with 791 traders in 18 Vietnamese live bird markets. Markets greatly differed according to the sources from which poultry was obtained, and their connections to other markets through the movements of their traders. These features, which could be informed based on indicators that are easy to measure, suggest that markets could be used as sentinels for monitoring virus strains circulating in specific segments of the poultry production sector. AIV spread within markets was modelled. Due to the high turn-over of poultry, viral amplification was likely to be minimal in most of the largest markets. However, due to the large number of birds being introduced each day, and challenges related to cleaning and disinfection, environmental accumulation of viruses at markets may take place, posing a threat to the poultry production sector and to public health.

In recent years, there has been increased diversity of avian influenza virus (AIV) subtypes infecting poultry populations in East and South-East Asia, including Vietnam^1^,^2^. Monitoring the circulating subtypes is essential, as these viruses can cause major poultry production losses and also pose a concern for global health security^3^. Conventional AIV surveillance systems relying on passive reporting of disease events by farmers are not appropriate in most countries where these viruses are endemic, as they will not rapidly capture new virus introductions or strain evolution. While some zoonotic subtypes, such as H7N9, do not seem to be pathogenic in poultry^4^, poultry infection by highly pathogenic subtypes may also remain unnoticed due to sub-optimal levels of flock vaccination^5^ or the reluctance of farmers to report disease outbreaks when facing the risk of uncompensated culling of their flock^6^. Instead, in order to improve the sensitivity of the surveillance programs, a risk-based approach informed by knowledge of the local factors influencing AIV dynamics must be adopted^3^.

The trade and marketing of live birds are known to be major pathways for AIV transmission^7^,^8^,^9^, with live bird markets (LBMs) being frequently found contaminated in AI-endemic settings^10^,^11^,^12^,^13^,^14^,^15^. The introduction of AIVs to LBMs may be promoted by the sourcing of birds from different sites, the visits of live bird traders to a large number of farms and LBMs, and a lack of biosecurity measures. The repeated introduction of infectious birds to LBMs will lead to the contamination of the LBM environment. If local conditions are suitable for the survival of viruses, AIVs may “accumulate” in the environment of these LBMs, and eventually spread to poultry farms through the movements of contaminated traders. LBMs also pose a public health risk, exposing traders and consumers to high loads of zoonotic pathogens. Moreover, depending on the management practices of their traders and, in particular, the time spent by birds in LBMs, virus circulation may even be amplified and sustained in LBMs^16^,^17^.

AIV surveillance has already been implemented in LBMs in several countries to monitor the genetic diversity of circulating strains^10^,^11^. However, if the objectives of surveillance programs are broadened to include tracing of infections detected in LBMs to the affected farming systems, or downstream to other markets or retail destinations as well as informing the design of control strategies, the selection of the participating LBMs and the design of surveillance activities must be based on a detailed study of the structure of the actual live bird trading and marketing systems. Trading practices influencing the risk of LBMs becoming contaminated, amplifying virus circulation and spreading it to other destinations need to be understood in order to interpret the findings of surveillance activities. Such knowledge would also allow a better allocation of resources so that LBMs associated with the highest likelihood of AIV detection can be identified and targeted. Likewise, such information would allow the selection of LBMs where the implementation of control interventions would be the most relevant, and to adapt these interventions to the LBM characteristics, optimising their effectiveness, while reducing their cost and limiting their disruptive impact on trading activities.

To address these knowledge gaps in Vietnam, a cross-sectional survey was conducted in LBMs in the north of the country. By way of structured interviews of live poultry traders, an assessment was made of the trading practices that are likely to influence the risk of AIVs being introduced into, and maintained within these LBMs.

## Results

**Figure 1:**
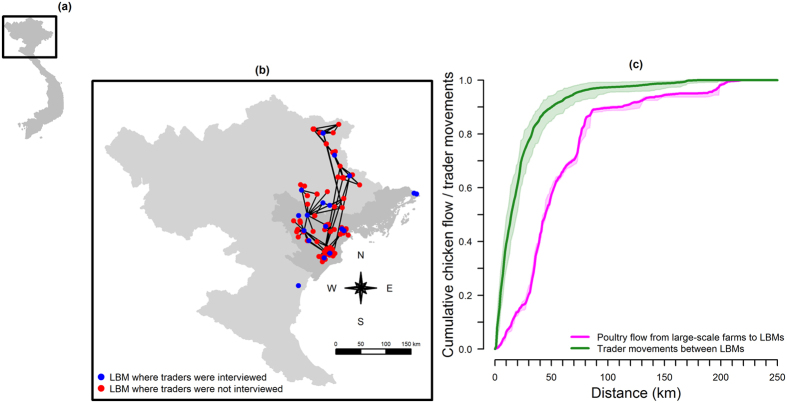
Study LBMs in northern Vietnam and distribution of distances travelled by traders. (**a**) Map of Viet Nam. (**b**) LBMs included in the survey (blue dots), LBMs identified through trader interviews (red dots) and trader journeys between LBMs (black lines) are shown. The Red river delta appears darker than the rest of northern Vietnam. (**c**) Cumulative distribution of poultry flow from large-scale farms to LBMs (in purple), and of trader journeys between LBMs (in green) are shown as a function of the distance. For both distributions, the median (line) and minimum and maximum (borders of the shaded area) are shown. Maps were generated using R 3.1.0^31^.

The study area included 13 provinces in northern Vietnam consisting of nine of 11 Red River delta provinces^18^ and four nearby provinces. The largest LBMs in the selected provinces in terms of the number of operating traders and the number of live poultry sold were identified. Eighteen LBMs (1–2 per province) were included in this study (blue dots on Fig. 1b), and a total of 791 traders were interviewed in these LBMs. The estimated number of traders operating in these LBMs on the day(s) that they were surveyed ranged from 7 to 110 (median: 59). In LBMs with 65 or less traders on the day(s) of survey, 92–100% of the traders were interviewed. In larger LBMs, at least 51% of traders were interviewed. The sale of broiler chickens, spent hens, and ducks, including Muscovy and Mallard-derived ducks, were reported. Of the interviewed traders, 80% (n = 631) sold chickens, while others sold ducks (10%, n = 82) or purchased but did not sell chickens (10%, n = 78). Spent hens were traded by less than 1% of interviewees. Duck sales are seasonal in contrast to chickens, and the survey did not take place during duck trading seasons. As the survey was consequently not able to capture duck trade patterns, we focused on chicken trade only. Further description of the trader population is given in the supplementary information.

### Number and sources of chickens sold

**Figure 2:**
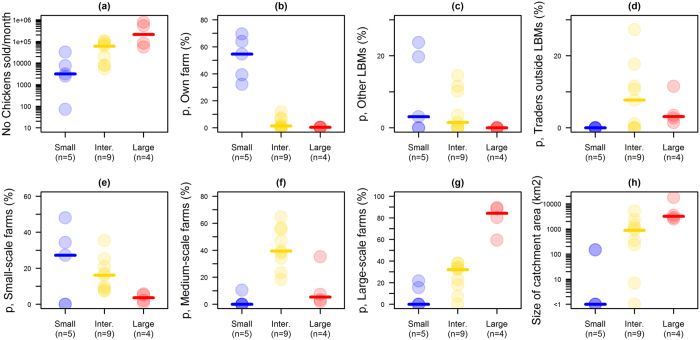
Estimated number of chickens sold in each LBM and their sources. The number of chickens sold and the proportion of chickens originating from each possible source are shown for each LBM according to their group: small (in blue), intermediate (in yellow) and large LBMs (in red). Some catchment areas (defined by the locations of a given LBM and its supplying large-scale farms) are equal to 0 as the survey only recorded the location of large-scale farms supplying LBMs.

In all but one LBM, more than 2,000 chickens were estimated to be sold per month, with a maximum of about 780,000 per month, and at least 71% of chickens offered for sale in the LBMs directly originated from farms. Based on the sources and numbers of chickens sold by their traders, LBMs were classified using principal component analysis (PCA) and hierarchical cluster analysis (HCA)^19^. LBMs were differentiated in 3 distinct groups (Fig. 2), named as “small”, “intermediate” and “large” LBMs. Note that the names of these groups referred the median number of chickens sold per LBM in each group. However, more chickens were sold in some small LBMs than in some intermediate LBMs, and more chickens were sold in some intermediate LBMs than in some large LBMs (Fig. 2a).

In small LBMs, most chickens were either sold by the farmers themselves, or were purchased by traders in small-scale farms (<50 chickens). Farmers or traders selling chickens from their own flock in the LBMs were mainly small-scale producers (97%, 158/163). The sources of chickens sold in intermediate LBMs were more diverse than for other LBM groups, with medium-scale farms (50–500 chickens) being the main source of chickens sold in 7 of the 9 intermediate LBMs. Contrary to other LBM groups, at least 60% of chickens sold in large LBMs originated from large-scale farms (>500 chickens).

The proportion of chickens supplied from other LBMs peaked at 24% in one small LBM, and was 0% for all large LBMs. All traders supplied by large-scale farms (n = 103) provided information about the locations of these farms. The size of the catchment area defined by the locations of a given LBM and its supplying large-scale farms was correlated to the number of chickens supplied by large-scale farms (Pearson’s correlation coefficient, *ρ* = 0.84), and peaked at 18,000 km^2^. Overall, half of the commercial movements of chickens from large-scale farms to the LBMs where they were sold occurred within a radius of 46 km around the LBMs (Fig. 1c). However, this distribution was over-dispersed. For 10% of these movements, the distance between a LBM and its supplying large-scale farms was estimated to be more than 110 km, with a maximum of 220 km.

### LBM networks

Of the interviewed traders, 21% (n = 164) visited at least two LBMs to sell and/or purchase poultry. In addition to the 18 surveyed LBMs, they reported operating in 119 other LBMs (Fig. 1b). Through the movements of all interviewed traders, 76% (n = 104) of these LBMs were connected to one another in a giant strong component (the largest subset of LBMs within which any LBM can be reached directly or indirectly by any other LBM following trader movements). Traders travelled shorter distances between LBMs than from large-scale farms to LBMs, with 50% of the movements between LBMs measuring less than 16 km. This distribution was also overdispersed, with 10% of trader movements between LBMs covering more than 50 km, up to 186 km.

**Figure 3:**
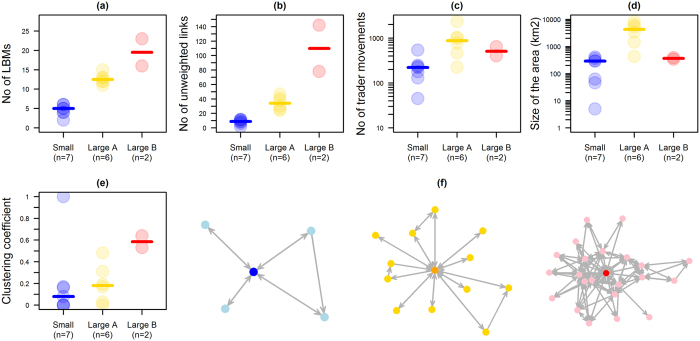
Features of egocentric networks. (**a–e**): Egocentric network features are shown for each LBM (excluding three isolated LBMs) according to their group: small (in blue), large (A; in yellow) and large (B; in red). (**f**): Examples of egocentric networks for each group.

For each surveyed LBM, egocentric networks were built based on trader movements between LBMs. As traders may mechanically carry and transmit viruses through the handling of birds or sharing of materials, it was assumed that LBMs were linked by the movements of their traders. These networks were directed and weighted, with the link strength being equal to the number of trader movements between two given LBMs over a one-month period. Three out of the 18 LBMs were isolated, meaning that all of their traders only operated in one LBM. Using PCA and HCA, the 15 other egocentric networks were classified according to their characteristics (see Methods). They were partitioned into three groups (small and large (A and B) networks) (Fig. 3). Small networks included the lowest number of LBMs and lowest number of trader movements between LBMs. The first group of large networks (Large A in Fig. 3) were characterized by the highest number of trader movements and extended over the largest geographical area. The second group of large networks (Large B in Fig. 3) included two networks which were characterised by the highest number of LBMs, up to 23, and unweighted links. Their clustering coefficients were also high; however, they extended over a small geographical area.

The number of traders operating in an LBM and also visiting other LBMs appeared to be a good predictor of the size of the corresponding egocentric network. Indeed, the number of traders visiting several LBMs was correlated to the number of nodes in a network (Pearson’s correlation coefficient, *ρ* = 0.87). When comparing the two partitions of LBMs according to (i) the number and sources of chickens sold, and (ii) their egocentric networks, it appeared that most LBMs classified as small were associated with large egocentric networks, whereas most LBMs classified as intermediate or large were associated with small egocentric networks (Table 1).

### Chickens and AIV dynamics in LBM

The time that chickens remained in each LBM was estimated based on the daily number of hours that traders reported to trade in a LBM, the frequency of supply, the frequency of having unsold chickens reoffered for sale the following day, and the number of such unsold chickens. A mathematical model was then used to estimate the value of *R*_0_ required for a virus to be amplified within a given LBM, given the observed chicken population dynamics. *R*_0_ was defined as the basic reproduction number – i.e. expected number of secondary cases from an average primary case in an entirely susceptible population^20^ – in an alternative closed population, of which the size was stable and no chickens left the population.

**Figure 4:**
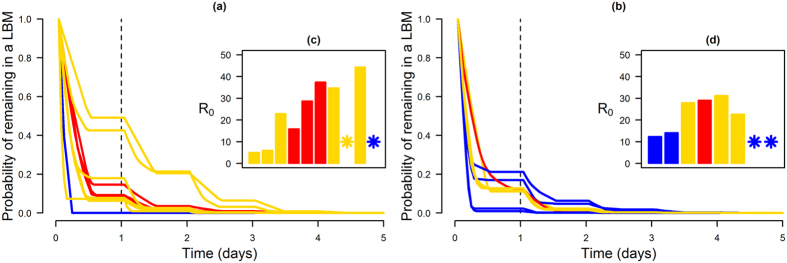
Time spent by chickens in LBMs and level of transmission required for virus amplification. The distribution of the time spent by chickens in each LBM before being sold are shown for LBMs associated with (**a**) small and (**b**) large egocentric networks. LBMs are differentiated into small (in blue), intermediate (in yellow) and large (in red) according to the number and sources of poultry sold. (**c,d**): the value of *R*_0_ required for virus amplification is shown for each LBM ordered by descending order to the probability of chickens remaining in a LBM for one day. *R_0_ > 50.

Figure 4 shows the probability that a chicken remained in each LBM as a function of time. In only two LBMs, the probability of chickens remaining there for more than 24 and 48 hours was higher than 0.21 and 0.06, respectively. Consequently, the level of AIV transmission required for an infected chicken to transmit the infection to more than one susceptible chicken was lower for these two LBMs than for other LBMs. Viral circulation could be amplified in these two LBMs for values of *R*_0_ – equivalent basic reproduction number in a closed population – above 5.4 and 6.4, respectively (Fig. 4c,d). These values were assessed assuming that 50% of transmission events (*ζ* = 0.5) were mediated by the environment, and the other 50% by direct contacts between chickens. Variations in *ζ* had a low impact on *R*_0_ estimates (supplementary information). While these two LBMs were classified as intermediate markets, they were among the seven LBMs selling the lowest quantity of chickens, and were associated with small egocentric networks. In contrast, in other intermediate and large LBMs, with or without associated large egocentric networks, the turn-over of chickens was higher, with chickens only remaining several hours in these LBMs before being sold. These LBMs were, therefore, unlikely to amplify viral circulation as the required level of transmission would be very high.

## Discussion

Surveyed LBMs were heterogeneous in terms of the practices of their traders. Most notably they greatly differed in the quantity of chickens sold. Without taking into account other factors, a large quantity of chickens sold would be expected to promote viral introduction into a LBM. It would also increase the likelihood of viral accumulation in the environment, due to increase in the number of infected chickens entering into the LBM and shedding viruses. Furthermore, it would increase the likelihood of viral amplification, as a larger population would reduce the risk of stochastic extinction of the circulating virus. However, while strong connections to the network of contacts between LBMs would be expected to increase the likelihood of a LBM being contaminated and further spreading the infection, the largest LBMs were not necessarily the most connected to other LBMs through the movements of their traders. Also, the origins of chickens sold in LBMs were shown to differ according to LBM size. Yet, the risk of a farm being contaminated and contaminating LBMs was likely to be influenced by its type and its location. For instance, the low level of biosecurity in small- and medium-scale farms^21^ may mean that their risk of infection was higher than for large-scale farms, which is from where most of the chickens sold in the largest LBMs originated. Therefore, the LBMs at the highest risk of contamination were not necessarily the largest LBMs. Their identification should be informed by further analyses accounting for the relative importance of the abovementioned contamination pathways. Nevertheless, this diversity of the farming systems and geographical areas supplying LBMs suggested that LBMs can be used as sentinels for monitoring AIVs circulating in specific segments of the poultry production system. In LBMs used as sentinels, the knowledge of trading practices and the results of disease surveillance activities could be used to inform further targeted surveillance activities aimed to track infections spreading in specific farming systems and other LBMs^22^.

Despite recruited LBMs being scattered throughout 13 provinces, most of them were connected to one another through the movements of traders. The short distance travelled by traders between LBMs and the features of egocentric networks suggest that the overall network of contact between LBMs was heterogeneous, highly connected (i.e. LBMs connected to the network are all included in a single component), with LBMs being preferably connected to others located in their vicinity. The predominance of local connections between LBMs may suggest spatial wave-like spread of viruses through this network. However, the small number of long-distance movements of traders may lead to the emergence of small-world properties and affect AIV dynamics through the network. Assessing the impact of the extent of trader movements on the structure of the network that they shape, and, therefore, the dynamics of diseases spreading through it, would need to be further studied.

It appeared that most LBMs with high number of sales and/or strong connections to other LBMs through the movements of their traders were associated with a high turn-over of chickens, limiting the potential for AIVs to be amplified and maintained there. The values of *R*_0_ required to amplify the virus were often higher than estimations available in the literature^23^,^24^. As the time spent by chickens in LBMs decreased, the expected time that infected chickens would spend in LBMs also decreased, offering less opportunity for transmitting the infection. Where the turn-over of the chicken population was high, amplification of viral circulation would, therefore, only be achieved for high values of *R*_0_. LBMs associated with a low likelihood of viral amplification and maintenance may, however, still pose substantial animal and public health risks. Indeed, depending on the prevalence of infection in farming systems supplying large LBMs, large numbers of infected birds may be frequently introduced into these LBMs, and viruses may “accumulate” in their environment. Viruses may then be transmitted from LBMs to farms through the movements of contaminated traders acting as fomites, as well as being a source of zoonotic infection for humans. Understanding such impact of poultry population dynamics on the potential for LBM to amplify viruses, or not, is necessary to inform sampling design and interpret surveillance results. In the absence or low likelihood of viral amplification, the absolute number of infected birds entering into large LBMs per day may be high, but the proportion of birds shedding viruses is likely to remain low. Under such a scenario, surveillance activities could be targeted towards the environment, in which viruses may “accumulate”, and be frequent due the limited time during which viruses may survive in these environments. In contrast, in LBMs where viruses are likely to be amplified by poultry, the prevalence of infection following viral introduction would be expected to be higher and the duration of viral circulation would be extended beyond the sale of birds that introduced the infection and the decay in infectiousness of the material they contaminated. There, sampling could target both the environment and birds, and could be less frequent than in LBMs where viral amplification is unlikely. Such understanding of poultry population dynamics in LBMs should also guide the choice of control interventions. Breaking the viral amplification cycle would require cleaning and disinfecting LBMs and slaughtering of unsold birds. In contrast, in LBMs associated with a high turnover of poultry, cleaning and disinfection alone would be sufficient. Due to the limited survival of the virus in the environment, these strategies would have to be applied very frequently, possibly daily, to significantly reduce the virus load accumulated in the environment^25^.

The study suggests that some indicators, which are easy to collect, may be good predictors of the level of interactions of a particular LBM with associated poultry farming systems and other LBMs. While the number of traders also operating in other LBMs was also found in another survey to be a good predictor of the position of a LBM in the network of contacts between LBMs^25^, the possible association between the number of poultry sold in a LBM and their sources would require further investigations. The small number of LBMs included in the current study indicates that caution needs to be applied when drawing conclusions from these results. Moreover, while 17 LBMs were visited for the entire period during which marketing activities took place on the day(s) of the interviews, the largest LBM in terms of chicken sales was not visited for the entire 24 hour period during which it was open. The number of traders, especially those spending only a couple of hours or even less time, was likely to have been underestimated, meaning that the quantity of chickens sold, their sources and the egocentric network structure might also have been incorrectly estimated. As interviews were carried out in each LBM during one or two days, it is possible that the number of observed traders was not representative of the number of traders usually operating there. There might also have been a recruitment bias towards traders operating in LBMs for several hours, compared with traders spending only a short time in LBMs. Such a bias may have impacted our LBM-level estimates.

In this survey, only information about the locations of large-scale farms was collected. The selection of medium- and small-scale farms, and especially the distances between those farms and the LBMs that they supply are likely to differ compared to large-scale farms. Moreover, the survey was cross-sectional, and it is unknown how the size of the catchment areas may vary over time. Repeated cross-sectional surveys conducted in China suggested that the locations of farms supplying traders operating in given LBMs varied from one month to the next^26^. However, the timescale and amplitude of these changes, as well as the possible impact of sampling error require further exploration. The lack of knowledge about the origin of poultry supplied to LBMs drastically limits the effectiveness of animal health service responses in face of an outbreak, and shows the need for a national farm registration and traceability system. Alternatively, understanding the factors influencing the selection of sources of chickens by traders would help in order to predict the catchment area of LBMs for which detailed information on trading practices are not available. Gravity modelling has been widely used in economics to predict the spatial movements of people and goods^27^. They have also been applied to epidemiology to assess the contribution of travels to disease spread between humans^28^. Adapted to the trade of live poultry, and informed by the results of further surveys, these models could be used to predict the commercial movements of poultry from producing areas to LBMs.

The AIV transmission model assumed homogeneous mixing, and time-constant transmission rate. However, some chickens are kept in cages at LBMs, and are, therefore, more likely to mix with chickens kept in the same cage, or at the same stall, rather than with chickens kept in distant stalls. Moreover, we assumed that chickens that were not offered for sale on the day they were introduced in a trader flock mixed with other chickens in LBMs, and that unsold chickens were also kept at the LBMs when the LBM was closed. However, some traders may keep recently purchased chickens not yet offered for sale, and chickens left unsold at the end of the day, at another location. For a substantial period of their stay in trader flocks, these chickens would then only mix with a fraction of the chicken population considered in the model. This clustering in mixing means that saturation in the actual transmission process is more likely to occur than predicted by our model, limiting viral amplification and promoting extinction. In LBMs, chickens are frequently handled, and moved between cages. Therefore, regardless of the number of chickens offered for sale, potentially infectious contacts between chickens or with contaminated fomites are more likely when LBMs are open rather than closed. The probability of direct and environment-mediated effective contacts within and between stalls is also very likely to vary according to the LBM setting. It would be impacted especially by the proximity between live poultry stalls, the frequency and effectiveness of cleaning and disinfection, and other hygiene measures, as well as poultry slaughtering activities taking place in the LBM. While a more complex meta-population model with time-varying transmission parameters would have better captured these factors impacting of AIV transmission dynamics in LBMs, our objective here was to identify LBMs where the time spent by chickens at the market created conditions promoting viral amplification.

In conclusion, the surveyed LBMs were heterogeneous in terms of trading practices influencing the risk of virus introduction and amplification. The variety of farming systems supplying them, and their connection to other LBMs suggested that they could be used as sentinels to monitor AIV circulation and evolution, and track infection spread, in identified segments of the poultry production system. While some LBMs may have an increased risk of virus introduction due to the high quantity of chickens they sold and/or their connection to other LBMs through the movements of their traders, they may have a reduced potential for amplifying and sustaining the virus circulation. Therefore, virus circulation may only be transitory in these LBMs and associated with a low level of prevalence, suggesting that, while surveillance could be targeted towards them, biological sampling design should account for these features in order for virus detection to remain sufficiently sensitive.

## Methods

### Selection of LBMs and live bird traders

A LBM was defined as an open space with at least two traders selling live poultry at least once per week and with official government authorisation to do so. The selection of LBMs was purposive. It was expected that most LBMs would sell only a very small number of chickens and be weakly connected to the network of live poultry trade, with only a small number of LBMs accounting for the majority of poultry sales and thereby playing an important role in AIV dynamics^25^. Therefore, the largest LBM in each of the 13 provinces, in terms of the number of operating traders and the number of live poultry sold, was selected. In 5 provinces, the two largest LBMs were expected to be of similar size and were both selected. These LBMs were identified through a workshop which involved officers from the sub-Departments of Animal Health within the Ministry of Agriculture and Rural Development from each of the 13 selected provinces. Preliminary visits to the identified LBMs were conducted in order to confirm the information provided during the workshop.

The traders considered to be part of the trader population of interest were all traders purchasing poultry within a given LBM with the aim to sell it at another location, and all traders offering poultry for sale within the given LBM or within a 500 meters radius. A minimum number of traders to be interviewed per LBM was defined in order to be able to identify most of the locations visited by traders (e.g. other LBMs or districts with poultry farms). Details regarding the sample size calculation are given in the supplementary information.

### Data collection

A questionnaire was designed for the interview of live bird traders. It was translated into Vietnamese and administered by trained interviewers. The questionnaire was piloted in LBMs which were not included in the survey. The visits to administer the questionnaires were scheduled according to information obtained during preliminary visits to each LBM about the days and times during which live birds were sold. In each LBM, traders were interviewed over either one or two days in January 2014. During the day(s) of interview, 17 out of 18 LBMs were visited for the entire period during which live bird marketing activities took place, in order to assess the number of traders operating there. The remaining LBM was not visited for the entire 24 hour period during which it was open. Informed oral consent was obtained from all participants prior to interviewing^16^,^29^. Questionnaires were reviewed immediately after the interviews by the head of the field team for missing or inconsistent information and, if required, traders were asked to clarify their responses. The study was approved by the Royal Veterinary College Ethics and Welfare Committee, and methods were carried out in accordance with the approved guidelines.

Traders were asked about practices which could influence the likelihood of (i) a LBM becoming contaminated, and (ii) a LBM amplifying viral circulation. The contamination of a LBM was assumed to be possible through the introduction of an infected bird or equipment contaminated by AIV. The main practices explored were the type, number and sources of poultry sold, and the frequency of visits to other LBMs. Specifically, traders were asked about the number of chickens sold in a given LBM within a day and the number of days during which they operated in this LBM in the preceding month. They were also asked about the proportion of their chickens which were sourced from the LBM where the interview was taking place, from other LBMs, from their own farm or other farms, or from other traders by the roadside. Farms were differentiated according to their size: small- (<50 chickens), medium- (50–500) and large-scale (>500). The names of the districts and provinces, of the large-scale farms supplying the traders were recorded. The names and locations of all LBMs that traders visited to purchase or sell poultry, as well as the number of days that they visited these LBMs during the previous month and the time of the visits, were also recorded.

The period of time that birds spent in the traders’ flocks, previously identified as a major factor influencing the potential of a LBM to amplify virus circulation^16^,^17^, was determined by the management of bird supply and surplus (surplus referring to the unsold poultry reoffered for sale the following day). Traders were asked about the frequency at which they purchase chickens, the average number of days during which they have surplus over a 10 day consecutive period, and the number of chickens constituting the surplus.

### Data analysis

Data were entered in duplicate and compared using SPSS surveycraft software (IBM). LBM features influencing the likelihood of contamination were described: the number and sources of chickens sold and the network of contacts with other LBMs which resulted from the movements of traders^25^. The LBM’s potential to amplify virus circulation was calculated based on the length of time that birds remained there. Since only a sample of traders operating in a given LBM was usually interviewed, the actual trader population operating in each LBM was simulated, as described below.

### Simulating the trader population operating in a LBM

The trader population operating in LBM *k* over a one-month period was simulated by re-sampling traders, using the following algorithm. First, the expected number of trader-days per month was calculated as the product between the number of traders observed operating in LBM *k* on the day of the survey and the number of days during which this LBM was open over one month. Secondly, the number of trader-days for the interviewed trader population *N*_*k*_ in LBM *k* was equal to 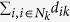, with *d*_*ik*_ the number of days that trader *i* offered chickens for sale over the previous month. Traders were randomly resampled individually from the pool of interviewed traders and added to the simulated trader population until 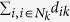 was equal to the expected number of monthly trader-days. This algorithm was repeated 1000 times, resulting in the simulation of 1000 populations of traders for each of the LBMs. The crude results obtained directly from interviewed traders without simulating the trader populations are provided in the supplementary information.

### Number and sources of chickens sold

The number *C*_*k*_ of chickens sold in a month by the *N*_*k*_ traders operating in LBM *k* was defined in equation (1):


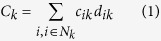


where *c*_*ik*_ was the daily number of chickens sold by trader *i* in LBM *k*. However, some traders reported purchasing chickens in the LBM where they were interviewed. If the traders who sold them the chickens were also interviewed, this formulation would lead to an overestimation of the actual number of chickens introduced into this LBM. An alternative formulation was therefore also used (equation (2)):


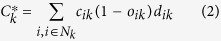


with *o*_*ik*_ being the fraction of chickens sold in a given LBM *k* that were actually purchased in the same LBM. Results obtained with the later formulation are reported in supplementary information.

The proportion *p*_*kh*_ of chickens sold in a LBM *k* that were obtained from a given source *h* (e.g. from other LBMs) was given by:


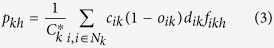


with *f*_*ikh*_ being the proportion of chickens sold by trader *i* in LBM *k* obtained from a source *h* (other than the LBM *k*).

The location of a LBM was assumed to be the centroid of the commune (a lower-level administrative division) where it was located. As traders reported only the district and/or province names where the supplying farms were located, farm locations were randomly sampled from within the boundaries of the reported districts or provinces. The estimation of the distribution of chicken flow as a function of the distance between a given LBM and the large-scale farms supplying it was based on 1000 simulations of farm locations. Likewise, the median size of the catchment area defined by the locations of a given LBM and its supplying farms was also estimated based on 1000 simulations.

Based on the median values of the aforementioned variables (number of chickens sold per month, proportion of chickens supplied from each possible source, size of the catchment area), LBMs were classified using principal component analysis (PCA) and hierarchical cluster analysis (HCA)^19^. PCA reduced the dimensions of the multivariate data by creating a small number of uncorrelated components, which are linear combinations of the initial variables and account for most of their variability. Using HCA, LBMs were then grouped according to their level similarity in these components. Similarity between two LBMs was measured by the Euclidian distance, and an agglomerative algorithm with a Ward’s criterion for linkage was applied.

### Live bird market networks

Networks of contacts between LBMs were constructed with LBMs as nodes and movements of traders as links. Egocentric networks were built for each LBM included in the survey, based on the movements of the simulated population of traders in the LBM. These networks were directed, e.g. a trader may go from market A to market B but not necessarily from B to A. The networks were also weighted, with the link strength being equal to the number of trader movements between two given LBMs over a one month period. The specific days that these LBMs were visited by each trader were unknown. They were therefore defined stochastically from the number of days that the traders reported visiting the LBMs within the previous month. For each set of traders and LBMs, 1000 stochastic egocentric networks were simulated. Further details about these simulations are provided in^25^. Each network was described using the following measures: number of nodes (i.e. LBMs), number of unweighted links between nodes (i.e. number of links between LBMs, accounting for their direction but not for the actual number of movements through each link), total number of trader movements, size of the geographical area defined by the network, and weighted clustering coefficient^30^. When the commune was known, the location of the LBM was taken as the centroid of the commune. Otherwise, the LBM’s location was randomly sampled within its district. A thousand simulations were conducted to assess the median size of the catchment area of these networks. Using PCA and HCA, LBMs were classified based on these variables. In addition, the overall network of contacts between LBMs by all traders and all identified LBMs was built. The size of the network’s giant strong component, the largest subset of nodes in which any node can reach any of the other nodes through direct or indirect network links, was assessed.

### Poultry population dynamics within LBMs

The distribution of the time spent by chickens in a given LBM was estimated through simulations, using the following algorithm. At time *t* = 0, a chicken was randomly allocated to a trader *i*, depending on the quantity of chickens sold by each trader. As not all traders were necessarily supplied with new chickens every day that they operated at a LBM, this meant that some chickens could be kept for one or more days before being offered for sale. The number of days that a chicken was kept before sale was generated through a multinomial process. For instance, if a trader purchased chickens on every second day that he operated at a LBM, the probabilities associated with chickens being stored *d* days before sale were *P*_*d*=0_ = *P*_*d*=0_ = 1/2 and *P*_*d*≥2_ = 0. If a trader was supplied every day, *P*_*d*=0_ = 1 and 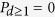, meaning that all its chickens were offered for sale from *t* = 0. Once offered for sale, the number of days that the chicken remained unsold was generated by a negative binomial process. The targeted number of successful trials was equal to one (i.e. the chicken was sold), and the probability of success was equal to 1 − *τ*_*i*_υ_*i*_, with *τ*_*i*_ the probability of trader *i* having a surplus, and υ_*i*_ the proportion of chickens left unsold when there was a surplus. On the day that the chicken was sold, the time of the sale was randomly picked from the uniform distribution [0, *T*_*ik*_], with *T*_*ik*_ being the length of time spent by a trader *i* in LBM *k* in one day. For each LBM, the distribution of the number of days spent by chickens in the LBM was assessed by conducting 100,000 iterations of this algorithm.

### AIV dynamics within LBMs

The spread of AIVs within a LBM was simulated using a stochastic discrete-time model, with an hourly time-step. Each day, all chickens entering into a LBM were introduced at the same time. Their probability to be sold in a given time-step was informed by the estimated distribution of the time spent by chickens in the LBMs (as described above). Chickens could successively move from the susceptible state, to the infected but not infectious state (i.e. pre-infectious), and then to the infectious state, resulting in death at the end of the infectious period. This transition between infection states is described in^17^. Briefly, the latent and infectious period of each bird was composed of a fixed and a stochastic integer, with the latter being generated by a binomial process. Infectious chickens contaminated their environment by releasing infectious faeces at each time-step, of which the infectiousness decreased exponentially over time. Therefore, a susceptible chicken could become infected through direct contact with infectious chickens or the contaminated environment. Density-dependence transmission and homogeneous mixing was assumed between all chickens present in an LBM at time *t*. The probability of a chicken becoming infected between *t* and *t* + *dt* was given by equation (4):


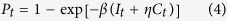


*I*_*t*_ was the number of infectious chickens and *C*_*t*_ the environmental load in the LBM at time *t*. *β* was the hourly rate of transmission and *η* the relative rate of transmission from the environment compared with *β*. *R*_0_ was defined as the basic reproduction number in an alternative closed population, of which the size *N*_0_ was stable and no chickens left the population. *β* and *η* were then expressed as:


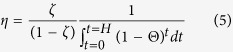



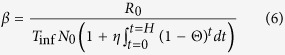


*ζ* denoted the proportion of transmission events mediated by the environment and *T*_inf_ the average infectious period. The integral accounted for the decay in infectiousness, at hourly rate 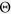, of faeces released by infectious chickens. Parameter values for AIV subtype H5N1 were used, and are presented in Table 2. Further details are provided in^17^.

For any given value of *R*_0_ and *ζ*, the basic reproduction number 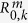 associated with LBM *k* could be estimated. 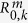 accounted for the population dynamics in an LBM *k*, and its calculation is detailed in the supplementary information. The values of *R*_0_ and *ζ* required to amplify viral circulation in a LBM (i.e. 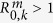) were calculated. All analyses were run using R 3.1.0^31^.

## Additional Information

**How to cite this article**: Fournié, G. *et al*. Investigating poultry trade patterns to guide avian influenza surveillance and control: a case study in Vietnam. *Sci. Rep.* **6**, 29463; doi: 10.1038/srep29463 (2016).

## Supplementary Material

Supplementary Information

## Figures and Tables

**Table 1:** Partition of live bird markets according to the number and sources of chickens sold and their egocentric networks.

	Isolated LBMs and small networks	Large networks (A)	Large networks (B)
Small LBMs	1 (6%)	2 (11%)	2 (11%)
Intermediate LBMs	6 (33%)	3 (17%)	0
Large LBMs	3 (17%)	1 (6%)	0

**Table 2:** AIV transmission model parameter values.

Parameter	Description	Value (unit)	Reference
d*t*	time-step	1 (hour)	
Θ	decay rate in faecal infectiousness per time-step	0.043	32
*H*	length of infectious period of faeces	96 (hours)	32
*T*_lat_	latent period	3 + *B*(8,3/8)d*t* (hours)	24
*T*_inf_	infectious period	43 + *B*(12,5/12)d*t* (hours)	24
