# Supplementary material for: Investigating poultry trade patterns to guide avian influenza surveillance and control: a case study in Vietnam
Source: Sci Rep. 2016 Jul 12;6:29463. doi: 10.1038/srep29463 (PMC4942603; doi:10.1038/srep29463)
Supplement: Supplementary Information [file srep29463-s1.pdf]

Supplementary Information

Title: Investigating poultry trade patterns to guide avian influenza surveillance and control: a case study in Vietnam

Authors: Guillaume Fournié, Astrid Tripodi, Nguyen Thi Thanh Thuy, Nguyen Van Trong, Tran Trong Tung, Andrew Bisson, Dirk U Pfeiffer, Scott H Newman

Description: Further details are provided about (i) algorithms for sample size calculation, (ii) algorithms for basic reproduction number calculation, (iii) distribution of trading practices, (iv) results of the multivariate analysis, (v) impacts of changes in the proportion of environmentally-mediated transmissions on basic reproduction number estimates.

1. Algorithm for sample size calculation

Using the following algorithm, the minimum number of traders to be interviewed per LBM was defined in order to identify with a probability of 95% at least 90% of the "sites" visited by traders to purchase or sell poultry, assuming that each trader visited 5 sites, and each site was visited on average by 5% of traders operating in that LBM.

A LBM with N traders was simulated. Each trader visited n_L sites, and each site was visited by a proportion P of the traders operating in that LBM. A site could be either another LBM or the poultry farms in a district (second administrative division).

First, the total number of sites L visited by the population of traders was calculated as:

$$L = n_L / \max\left(1/N, P\right)$$

The formulation of the denominator ensures that it lied between $1/N$ (each site was visited by one trader only) and 1 (each site was visited by all traders). L was rounded to the nearest integer.

For each trader, n_L different sites were randomly drawn. Although the L sites were visited by an average of NP traders, the random allocation of these sites meant the actual number of traders visiting each site varied.

s traders were then randomly sampled. The proportion p of sites visited by traders operating in this LBM that were identified through this sample was computed.

This algorithm was repeated 10,000 times, and the proportion of simulations for which p was equal or higher than a defined threshold T was assessed.

Here, n_L=5, P=0.05 and T=0.9. Sample sizes for which the probability to detect at least a proportion T of all sites visited by traders was higher than 0.95 are reported in Supplementary Table S1.

Supplementary Table S1: Sample size as a function of the size of the trader population in a LBM. It was calculated to detect, with a probability of 95%, 90% of all sites visited by traders operating in a given LBM to purchase or sell poultry, assuming that 5 sites are visited by each trader, and each site is visited by an average of 5% of traders.

Number of traders	Sample size
<20	All
30	27
40	34
50	40
70	48
100	53
>100	53

2. Basic reproduction number calculation

The dominant eigenvalue of the next generation matrix was the estimate of the basic reproduction number $R_{0,k}^{m}$ in LBM k [1]. Each element $r_{z,x}$ of the matrix was the expected number of chickens becoming pre-infectious after having spent x hours in the LBM due to direct or

indirect contacts with a primary case that became pre-infectious after having spent *z* hours in the LBM. It was given by:

$$r_{z,x} = \beta N P_S\left(t = x\right) \sum_{y, y \in Y} \left[\Omega\left(t = y \mid t_P = z\right) + \eta \Psi(t = y \mid t_P = z)\right]$$

β was the transmission rate. N was the size of a cohort (i.e. the number of chickens entering into a LBM on a given day), and $P_S\left(t = x\right)$ the probability that a chicken was still in the LBM after *x* hours. As specified in the Methods, all chickens introduced on the same day were introduced at the same time, and the timing of introductions was the same each day. *Y* was the set of values of *t* which were coincidental to *x*, i.e. corresponded to the same time of the day as *x*. For instance, if *x*=4, then $Y = \left\{4, 28, 52, 76, ...\right\}$. *Y* was therefore the set of *t* values for which the remainder of their Euclidian division (i.e. modulo operator) by 24 was the same as for *x*.

$\Omega\left(t = y \mid t_P = z\right)$ referred to the direct transmission process, through contacts with the primary case:

$$\Omega\left(t = y \mid t_P = z\right) = P_S\left(t = y \mid t_P = z\right) P_I\left(t = y \mid t_P = z\right)$$

With $P_S\left(t = y \mid t_P = z\right)$ the probability that a chicken becoming pre-infectious at time $t_P = z$ was still in the LBM at $t = y$, and $P_I\left(t = y \mid t_P = z\right)$ the probability that it was infectious at time $t = y$, given that it was still in the LBM. Note that if $z > y$, then $P_S\left(t = y \mid t_P = z\right) = P_I\left(t = y \mid t_P = z\right) = 0$.

$\Psi(t = y \mid t_P = z)$ referred to the indirect transmission process, through contacts with the contaminated environment. It accounts for infectious faeces released each hour by the primary case, from the onset of infectiousness at time $t = h$ to the time $t = y$ at which exposure occurs.

$$\Psi(t=y \mid t_P=z)=\sum_{h,h\geq z}^{y} P_S\left(t=h \mid t_P=z\right) P_I\left(t=h \mid t_P=z\right)\left(1-\Theta\right)^{y-h}$$

Θ is the decay rate in faeces infectiousness per hour.

The calculation of R_0^m was checked numerically. As expected, the probability of viral invasion in LBMs increased sharply around $R_0^m = 1$ (Supplementary Fig. S1).

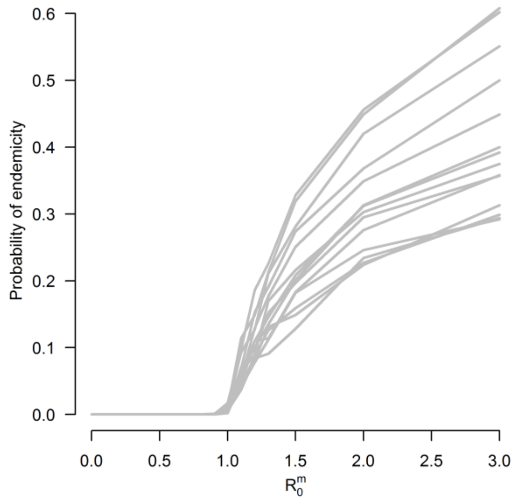

Supplementary Figure S1. The probability of disease endemicity as a function of R_0^m. This probability was assessed through simulations for the demographic profiles associated to the 14 LBMs which required R_0 to be lower 50 for R_0^m to be higher than 1. For each simulation, a bird was initially set as infected, and the number of susceptible birds entering into markets every day was chosen to be high (10,000) in order to avoid extinction once the disease reached its endemic equilibrium. Therefore, in this specific setting, a viral introduction resulting in a major outbreak in the host population would also lead to the disease becoming endemic – disease invasion was here similar to disease endemicity. The probability of disease endemicity (or invasion), for a given demographic profile, was the proportion of simulations for which at least one bird was infected or virus was present in the environment 100 days after viral introduction.

3. Description of the multivariate analysis results

The PCA was performed for LBMs based on variables related to (i) the number and sources of chickens sold, and (ii) the egocentric network characteristics. For both analyses, the two first components were selected. For the former analysis, the two first components accounted for 40% and 20% of the data variability, and for the latter analysis they accounted for 46% and 23%. LBMs with a high score for each of these components are described below. The converse is true for LBMs with a low score.

The number and sources of chickens sold

Component 1: LBMs sold large number of chickens which were mainly supplied by large-scale farms covering a large geographical area. Supply originating from small-scale farms, trader own farms, and other markets was low.

Component 2: traders mainly sold chickens from their own farm, and no birds were brought from other LBMs.

Structural characteristics of egocentric networks

Component 1: The egocentric networks included a large number of LBMs and a high number of unweighted links.

Component 2: The egocentric networks were characterised by a high number of trader movements, covered a large geographical area and had a low level of clustering.

4. Practices of interviewed traders

The practices of all interviewed traders are summarised in Supplementary Table S2.

5. Partitions of LBMs under different assumptions

LBMs were partitioned according to the number and sources of chickens sold under different assumptions: crude (Supplementary Tables S3-S4) or simulated trader populations (Supplementary Tables S5-S6), with the calculation of the number of chickens sold accounting (Supplementary Tables S3-S5) or not (Supplementary Tables S4-S6) for the number of chickens purchased in the LBM. The partition of LBMs into three groups (small, intermediate and large) was not affected by these assumptions.

The partitions of egocentric networks using crude and simulated trader populations differed by one LBM. It was assigned to Large (B) group when using crude trader populations (Supplementary Table S7), and to Large (A) group when using simulated trader populations (Supplementary Table S8).

Supplementary Table S2. Practices of interviewed traders. Data are median (inter-quartile range), or no (%); median and IQR are computed only for traders reporting the described practice; no (%) only accounts for traders who reported selling in the visited LBM, except for "visiting other LBMs" (all traders accounted for); *proportion of chickens sold by a trader that are obtained from the given source.

	Variable	Values
Sales	no of chickens sold/month	450 (100-1450)
Presence at LBM of interview	no days/months	20 (10-30)
Supply, source of chickens		
LBM of interview	no of traders	73 (12%)
	prop. of chickens sourced*	100% (100%-100%)
Other LBMs	no of traders	40 (6%)
	prop. of chickens sourced*	100% (79%-100%)
Other traders (outside LBMs)	no of traders	38 (6%)
	prop. of chickens sourced*	100% (100%-100%)
Farms	no of traders	447 (71%)
	prop. of chickens sourced*	100% (100%-100%)
Trader's own farm	no of traders	163 (26%)
	prop. of chickens sourced*	100% (100%-100%)
Small-scale farms	no of traders	122 (19%)
	prop. of chickens sourced*	50% (30%-100%)
Medium-scale farms	no of traders	156 (25%)
	prop. of chickens sourced*	80% (50%-100%)
Large-scale farms	no of traders	103 (16%)
	prop. of chickens sourced*	100% (95%-100%)
Supply frequency: every time	no of traders	524 (66%)
Supply frequency: not every time	no of traders	267 (34%)
Visiting other LBMs	no of traders	164 (21%)
	no of visited LBMs	2 (2-3)
Reporting surplus	no of traders	453 (72%)
	reported frequency	0.3 (0.2-0.4)
	prop. unsold chickens	0.2 (0.2-0.4)

Supplementary Table S3. Partition of LBMs according to the number and sources of chickens sold using crude trader populations and accounting for the number of chickens purchased in LBM. For each variable, the median, minimum and maximum values are shown.

	All	Small LBMs	Inter. LBMs	Large LBMs
N	18	5	9	4
number of chicken sold/month	23,912 (25-275,025)	2760 (25-3862)	31,775 (4070-66,553)	138,544 (50,222-275,025)
percentage, other LBMs	0 (0-24)	3 (0-24)	1 (0-14)	0 (0-0)
percentage, traders (roadside)	1 (0-29)	0 (0-0)	8 (0-29)	3 (2-12)
percentage, own farm	2 (0-69)	54 (32-69)	2 (0-12)	0 (0-0)
percentage, small-scale farms	9 (0-48)	28 (0-48)	16 (7-35)	4 (1-6)
percentage, medium-scale farms	21 (0-65)	0 (0-11)	39 (18-65)	5 (2-35)
percentage, large-scale farms	28 (0-89)	0 (0-22)	32 (0-38)	84 (59-89)
Size catchment area (km^2)	612 (0-17,963)	0 (0-150)	874 (0-5187)	3202 (2490-17,963)

Supplementary Table S4. Partition of LBMs according to the number and sources of chickens sold using crude trader populations and ignoring the number of chickens purchased in LBM. For each variable, the median, minimum and maximum values are shown.

	All	Small LBMs	Inter. LBMs	Large LBMs
N	18	5	9	4
number of chicken sold/month	25,792 (25-407,525)	2760 (25-3862)	35,535 (4820-69,403)	153,791 (53,040-407,525)
percentage, other LBMs	0 (0-24)	3 (0-24)	1 (0-14)	0 (0-0)
percentage, traders (roadside)	1 (0-29)	0 (0-0)	8 (0-29)	3 (2-12)
percentage, own farm	2 (0-69)	54 (32-69)	2 (0-12)	0 (0-0)
percentage, small-scale farms	9 (0-48)	28 (0-48)	16 (7-35)	4 (1-6)
percentage, medium-scale farms	21 (0-65)	0 (0-11)	39 (18-65)	5 (2-35)
percentage, large-scale farms	28 (0-89)	0 (0-22)	32 (0-38)	84 (59-89)
Size catchment area (km^2)	612 (0-17,963)	0 (0-150)	874 (0-5187)	3202 (2490-17,963)

Supplementary Table S5. Partition of LBMs according to the number and sources of chickens sold using simulated trader populations and accounting for the number of chickens purchased in LBM. For each variable, the median, minimum and maximum values are shown.

	All	Small LBMs	Inter. LBMs	Large LBMs
N	18	5	9	4
number of chicken sold/month	42,139 (70-525,350)	3128 (70-32,432)	59492 (4670-103,432)	280,100 (51,847-525,350)
percentage, other LBMs	0 (0-23)	3 (0-23)	1 (0-14)	0 (0-0)
percentage, traders (roadside)	1 (0-27)	0 (0-0)	8 (0-27)	3 (1-12)
percentage, own farm	2 (0-70)	55 (32-70)	1 (0-12)	0 (0-0)
percentage, small-scale farms	9 (0-48)	27 (0-48)	17 (7-35)	4 (1-6)
percentage, medium-scale farms	21 (0-65)	0 (0-11)	39 (18-65)	5 (2-35)
percentage, large-scale farms	27 (0-90)	0 (0-22)	32 (0-38)	84 (60-90)
Size catchment area (km^2)	612 (0-17,963)	0 (0-150)	874 (0-5187)	3202 (2490-17,963)

Supplementary Table S6. Partition of LBMs according to the number and sources of chickens sold using simulated trader populations and ignoring the number of chickens purchased in LBM. For each variable, the median, minimum and maximum values are shown.

	All	Small LBMs	Inter. LBMs	Large LBMs
N	18	5	9	4
number of chicken sold/month	43,732 (72-781,175)	3119 (72-32,442)	60,725 (5420-105,890)	308,439 (55,023-781,175)
percentage, other LBMs	0 (0-24)	3 (0-24)	1 (0-15)	0 (0-0)
percentage, traders (roadside)	1 (0-27)	0 (0-0)	8 (0-27)	3 (1-12)
percentage, own farm	2 (0-70)	55 (32-70)	1 (0-12)	0 (0-0)
percentage, small-scale farms	9 (0-48)	27 (0-48)	16 (7-35)	4 (1-6)
percentage, medium-scale farms	21 (0-65)	0 (0-10)	39 (18-65)	5 (2-35)
percentage, large-scale farms	28 (0-90)	0 (0-22)	32 (0-38)	84 (59-90)
Size catchment area (km^2)	612 (0-17,963)	0 (0-150)	874 (0-5187)	3202 (2490-17,963)

Supplementary Table S7. Partition of LBM egocentric networks using crude trader populations. For each variable, the median, minimum and maximum values are shown.

	All	Small networks	Large A networks	Large B networks
N	15	7	5	3
no of LBMs	6 (1-23)	5 (2-6)	13 (11-15)	16 (12-23)
no of unweighted links	11 (0-137)	9 (2-11)	36 (24-42)	76 (29-137)
no of traders' movements	150 (0-1368)	123 (44-455)	246 (114-406)	562 (353-1368)
size of the area (km^2)	353 (0-8387)	290 (5-412)	5426 (1444-8387)	390 (343-428)
clustering coefficient	0 (0-1)	0 (0-1)	0 (0-0)	1 (0-1)

Supplementary Table S8. Partition of LBM egocentric networks using simulated trader populations. For each variable, the median, minimum and maximum values are shown.

	All	Small networks	Large A networks	Large B networks
N	18	7	6	2
no of LBMs	6 (1-23)	5 (2-6)	12 (11-15)	20 (16-23)
no of unweighted links	12 (0-142)	9 (2-12)	34 (24-47)	110 (78-142)
no of traders' movements	241 (0-2334)	222 (45-540)	883 (223-2334)	520 (401-640)
size of the area (km^2)	353 (0-8387)	290 (5-412)	4426 (428-8387)	366 (343-390)
clustering coefficient	0 (0-1)	0 (0-1)	0 (0-0)	1 (1-1)

6. Impact of the proportion of environment-mediated transmission on R_0 estimates

Changes in ζ did not have any major impact on the value of R_0 required for amplifying viral circulation. For most LBMs, as ζ increases, the required value of R_0 slightly decreases (Supplementary Fig. S2).

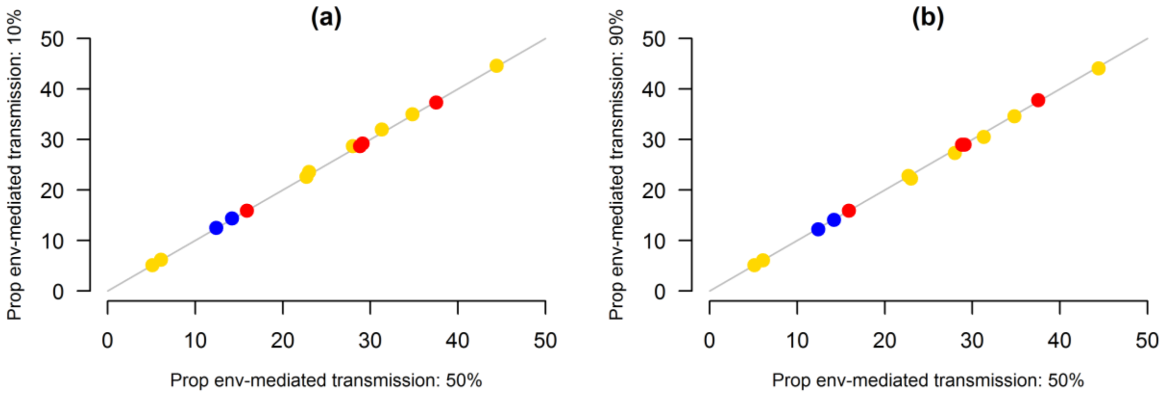

Supplementary Figure S2. The impact of variations of environment-mediated transmission on R_0 estimates. x- and y-axes are the values of R_0 required for amplifying viral circulation for different proportion of environmentally-mediated transmission.

References

1 Diekmann, O. & Heesterbeek, J. A. *Mathematical Epidemiology of Infectious Diseases : Model Building, Analysis and Interpretation*. (John Wiley & Sons, 2000).
